# Comprehensive quality of elderly rehabilitation nursing staff in medical and health care institutions in Liaoning Province, China: a cross-sectional study

**DOI:** 10.1186/s12877-022-03092-6

**Published:** 2022-05-10

**Authors:** Yunru Zhou, Longfeng Sun, Yanting Liang, Guoju Mao, Pei Xu

**Affiliations:** The First Hospital of China Medical University, Shenyang, Liaoning Province China

**Keywords:** Comprehensive quality, The combination of medical and health care, Elderly rehabilitation nursing

## Abstract

**Background:**

With the global aging problem is becoming increasingly severe, the elderly care has become an important issue that needs attention. Chinese government attaches great importance to the development of medical and health care institutions, and is committed to improving the comprehensive quality of elderly rehabilitation nursing staff in medical and health care institutions.

**Methods:**

From June to September 2019, a cross-sectional study among 193 elderly rehabilitation nursing staff was conducted in Liaoning Province, China. Using a self-designed questionnaire, the comprehensive quality of elderly rehabilitation nursing staff in medical and health care institutions was investigated by face to face. The multiple linear regression model was explored to analyze the influencing factors.

**Results:**

A total of 193 questionnaires were distributed, and 189 (97.93%) valid questionnaires were recovered. Age was from 19 to 65 years old, with an average age of (38.34 ± 9.76) years old. Bachelor degree or above accounted for 54.00%. 57.10% have engaged in elderly rehabilitation nursing for more than one year. There were 163 nurses with qualification certificates, accounting for 86.20%. The total score of comprehensive quality was 118.52 ± 22.90. The total Cronbach ' s α coefficient of the questionnaire was 0.967, and the content validity index was 0.991. Only 61 (32.30%) elderly rehabilitation nurses received professional training in elderly rehabilitation nursing. The results of multiple linear regression analysis showed that the educational level of elderly rehabilitation nursing staff (*P* = 0.002) and the number of years engaged in elderly rehabilitation nursing (*P* = 0.005) were the main influencing factors of comprehensive quality.

**Conclusions:**

The comprehensive quality of elderly rehabilitation nursing staff is at a medium level in Liaoning Province's medical and health care institutions. However, the professional nursing talents were very short, and the education level and years of experience in elderly care were the main influencing factors of the comprehensive quality.

## Background

At present, the aging problem is a universal problem in the world. In China, it is expected that by 2025, aging population will exceed 300 million [1]. The aging level of the eastern, central, western and northeastern regions increased synchronously in the unbalanced [2]. Since 2010, the proportion of people aged 65 and above in the northeast region has increased sharply, and gradually become the ' oldest ' region in China [3]. Elderly care has become an urgent problem to be solved. In 2015, the Health and Family Planning Commission and other departments indicated that there was an urgent need to provide the elderly with services that combined medical care and health care [4]. In 2019, the State Council clearly pointed out to build a comprehensive home-based, community-based, institution-supported functional, appropriate scale, covering urban and rural old-age service system by 2020 [5], which was a combination mode of medical and health care.

The combination mode of medical and health care is a pension mode that integrates pension, medical care, nursing, rehabilitation and hospice care. Its connotation is to organically combine the resources of medical care and pension, and integrate daily care, medical care, nursing and rehabilitation to jointly solve the problem of pension [6]. Judging from the current aging situation and the development trend of the elderly care industry in China, the combination of medical and health care is the general trend [7]. As early as the middle of the twentieth century, some developed countries abroad began the reform of the combination of health services and social care services for the elderly, similar to the reform of the combination of medical and health care in China. The typical representative examples are long-term acute care (LTAC) and home of long-term care (LTCH) comprehensive health care model for the elderly in the United States, Long-term care insurance system in Japan and comprehensive care model in Britain [8]. Since Guo Dong et al. [9] first proposed the mode of combination of medical and health care in 2005, the theory and practice of the mode have begun in China. At present, there are four modes in China, 1) carrying out elderly care services in medical and health care institutions, 2) adding medical services in pension institutions, 3) promoting the extension of medical and health services to the community or family to provide door-to-door services for the elderly, 4) the agreement cooperation between medical institutions and pension institutions [10]. Compared with the traditional daily care service for the elderly, the combination of medical and health care is more concerned about the needs of the elderly for medical health [11]. Therefore, the medical and health care institutions should not only provide basic daily care for the elderly living in the institutions, but also provide medical treatment and nursing, rehabilitation nursing, psychological intervention and health management.

As the government vigorously promotes the development of the combination of medical and health care, it has now been widely recognized by all sectors of society [12]. The results of Wei et al. [13] showed that the demand for daily care and medical care of the elderly was also much higher than that of other age groups. The elderly not only cared about their own health, but also more concerned about the quality of care in medical institutions [14]. However, Rezaei et al. demonstrated a low number of elderly rehabilitation nursing staff, insufficient training of nurses in this area, and a lack of clear understanding of the multidimensional needs of frail older adults [15]. Therefore, it is essential to comprehensively improve the comprehensive quality of elderly rehabilitation nursing staff in medical and health care institutions. At present, the researches on the combination of medical and health care focused on the development of the institution and the needs of the elderly lived in the institution. However, there are few studies on the comprehensive quality of the elderly rehabilitation nursing staff.

With a self-designed comprehensive quality status questionnaire, this study is to analyze the comprehensive quality status of elderly rehabilitation nursing staff in medical and health care institutions in Liaoning Province, China. We compared the comprehensive quality of different types of elderly rehabilitation nursing staff, and analyzed its influencing factors, which could help to improve the existing training programs, enhance the professional abilities of elderly rehabilitation nursing staff, and improve the service quality of elderly rehabilitation nursing staff and quality of life of the elderly living in the institutions.

## Methods

### Study design and participants

The design of the study was a cross-sectional study. We selected the participants who participated in the training course for elderly rehabilitation nursing staff in Liaoning Province, China from June 2019 to September 2019 in the First Affiliated Hospital of China Medical University, and conducted a face to face survey using a self-designed questionnaire.

### Eligibility criteria

Inclusion criteria: Elderly rehabilitation nursing staff who aged over 18 years old and worked in medical and health care institutions.

Exclusion criteria: Incomplete questionnaire.

### Procedures

The questionnaire of the comprehensive quality of the elderly rehabilitation nursing staff in medical and health care institutions was based on the relevant literature [16, 17], and developed after three rounds of expert consultation. According to the results of expert consultation, the content validity was calculated and expressed by Index of Content Validity (CVI). The item-level CVI (I-CVI) range was 0.800–1.000, of which only two items had the CVI value of 0.800, and the rest were 1.000. The score of the average scale-level CVI / Ave (S-CVI / Ave) was 0.991, indicating that the questionnaire had good content validity. Factor analysis was used to test the structural validity of the questionnaire. The KMO value was 0.937, and the X^2^ value of Bartlett’ s spherical test was 7770.195 (*P* < 0.05), which was suitable for factor analysis. Using the maximum variance orthogonal rotation, the cumulative variance explained by the questionnaire was 69.678%, indicating that the structure validity was good. The Cronbach ' s α coefficient of the questionnaire was 0.967, with high internal consistency.

### Outcome and measures

The questionnaire of the comprehensive quality of the elderly rehabilitation nursing staff in medical and health care institutions includes two parts: 1) General information: such as gender, age, educational level, technical title, etc. 2) Comprehensive quality status: divided into professional knowledge, professional skills, professional attitude three dimensions, 47 items. The Likert 4 score method was used to assign 1–4 points from ' completely unknown ' to ' very understanding ', and the total score was 47–188 points. With a higher score indicating a higher level of comprehensive quality.

### Data collection

The research questionnaire of this study has passed three rounds of expert consultation stage. We issued questionnaires for the research objects, and explained the purpose, significance and methods of the research. After obtaining informed consent, the research objects filled out the questionnaires themselves. In the process of filling out, we answered the questions raised by the subjects, and emphasized the anonymous way to ensure the authenticity and accuracy of the survey results. Upon completion of the questionnaire, on-site recovery.

### Ethics approval and consent to participate

The experimental protocol was established, according to the ethical guidelines of the Helsinki Declaration. We confirmed that all experiments were approved by the Ethics Committee of the First Affiliated Hospital of China Medical University, all methods were performed in accordance with the Strobe statement. All participants were over 18, we confirmed that written informed consent was obtained from all participants themselves.

### Statistical analyses

The data obtained from the survey were checked and entered into Epidata3.1 by two people, and SPSS 22.0 was used for statistical analysis. Descriptive statistics were performed with mean ± SD or percentage (%), t test and one-way ANOVA were used for comparison between groups (LSD method was used for pairwise comparison). Multivariate linear regression analysis was used for influencing factor analysis. *P* < 0.05 indicated that the difference was statistically significant.

## Results

### Sociodemographic characteristics and bivariate analysis of elderly rehabilitation nursing staff in medical and health care institutions

A total of 193 subjects were investigated in this study, 189 subjects were included, and 4 subjects were not included because the questionnaire was incomplete and could not be used as valid data. 189 cases of elderly rehabilitation nursing staff, male 7 cases, female 182 cases. Age was 19–65 years old, with an average age of (38.34 ± 9.76) years old. 110 (58.20%) were from public general hospitals. Education level: 5 cases (2.60%) of junior high school and below, 34 cases (18.00%) of senior high school or technical secondary school, 48 cases (25.40%) of junior college, 102 cases (54.00%) of bachelor degree or above. 57.10% have engaged in elderly rehabilitation nursing for more than one year. There were 163 nurses with qualification certificates, accounting for 86.20%. (Table 1).

**Table 1:** Differences in characteristics of elderly rehabilitation nursing staff in medical and health care institutions

Variables	N	Percent	Mean ± SD	F/t	P
Gender					
Male	7	3.70	89.43 ± 20.69	-3.54	0.001
Female	182	96.30	119.70 ± 22.28		
Age (years)					
≤ 35	76	40.20	122.25 ± 23.25	3.58	0.030
36–55	100	52.90	114.62 ± 22.22		
≥ 56	13	6.90	127.62 ± 21.52		
Marital status					
Single	34	18.00	118.62 ± 24.13	0.42	0.660
Married	150	79.40	118.27 ± 22.46		
Divorced	5	2.60	127.80 ± 30.78		
Health status					
Health	178	94.20	119.54 ± 22.76	2.35	0.020
Chronic diseases or other	11	5.80	103.00 ± 20.34		
Education					
Junior high school and below	5	2.60	94.00 ± 14.88	10.25	< 0.001
Senior high school or technical secondary school	34	18.00	105.18 ± 22.89		
Junior college	48	25.40	116.27 ± 22.04		
Bachelor degree or above	102	54.00	125.34 ± 20.77		
Ownership of working unit					
Nursing home	36	19.00	111.92 ± 23.24	4.65	0.004
Community hospital	27	14.30	107.89 ± 16.79		
Geriatric hospital	16	8.50	121.88 ± 26.56		
Public general hospital	110	58.20	122.91 ± 22.44		
Monthly income (CNY)					
Less than 2000	16	8.50	107.25 ± 23.16	4.83	< 0.001
2000–4000	105	55.50	115.37 ± 22.49		
4001–6000	45	23.80	125.91 ± 20.95		
6001–8000	18	9.50	131.00 ± 18.60		
8001–10,000	3	1.60	131.33 ± 22.59		
Over 10,000	2	1.10	82.00 ± 16.97		
Years of elderly rehabilitation nursing					
Less than 1 year	81	42.90	113.11 ± 22.49	3.82	0.011
1–5 years	75	39.70	120.43 ± 21.80		
6–10 years	14	7.40	124.57 ± 24.68		
Over 10 years	19	10.00	130.21 ± 22.74		
Nurse practicing certificate					
No	26	13.80	96.81 ± 19.48	5.63	< 0.001
Yes	163	86.20	122.06 ± 21.49		
Technical title					
No	26	13.80	96.81 ± 19.48	9.16	< 0.001
Nurse	33	17.40	117.00 ± 24.30		
Senior nurse	50	26.50	119.94 ± 23.38		
Supervisor nurse	55	29.10	124.78 ± 18.38		
Co-chief superintendent nurse and above	25	13.20	118.58 ± 22.90		

By further comparison, it was showed that the comprehensive quality of elderly rehabilitation nursing staff in the age group of 35 years and below was significantly higher than that in the age group of 36–55 years (*P* < 0.05). The comprehensive quality of elderly rehabilitation nursing staff in public general hospital group was higher than that in nursing home group and community hospital group (*P* < 0.05). The comprehensive quality of elderly rehabilitation nursing staff in the bachelor degree or above education group was significantly higher than that in other education groups, and the differences were statistically significant (*P* < 0.05). In terms of technical titles, the comprehensive quality of the group without professional titles was significantly lower than that of other groups, and the differences were statistically significant (*P* < 0.01). The comprehensive quality of nursing staff engaged in elderly rehabilitation nursing for 1–5 years and more than 10 years was significantly higher than that of nursing staff engaged in less than 1 year (*P* < 0.05).

### Current situation of comprehensive quality of elderly rehabilitation nursing staff in medical and health care institutions

Comprehensive quality score of elderly rehabilitation nursing staff in medical and health care institutions, the maximum score was 182 points, and the minimum was 64 points. The mean score was 2.52 out of 4 (Min = 1.36, Max = 3.87), and the standard deviation was 0.54. The comparison of the scores in the three dimensions of comprehensive quality is shown in Table 2.

**Table 2:** Descriptive analysis of comprehensive quality and its subscales

Total	Professional Knowledge	Professional Skills	Professional attitude
2.52 ± 0.49	2.28 ± 0.54	2.55 ± 0.71	3.45 ± 0.28

### The training status of elderly rehabilitation nursing staff in medical and health care institutions

Among the 189 elderly rehabilitation nurses surveyed, only 61 had received professional training in elderly rehabilitation nursing, accounting for 32.30%. Table 3 shows the results of a survey of 61 nurses trained in elderly rehabilitation nursing. 60.00% of the 61 elderly rehabilitation nurses who participated in the training believed that the knowledge of specialized disease nursing training was the most important, and the knowledge of daily life nursing, mental and psychological nursing were also important.

**Table 3:** The training status of elderly rehabilitation nursing staff in medical and health care institutions

Categories	Options	%
Training cumulative time (days)	1–2	14.80
	3–7	44.30
	8–15	9.80
	16–30	13.10
	> 30	18.00
Training methods	Short-term pre-service centralized training organized by pension institutions	17.30
	Centralized training organized by civil affairs departments	12.50
	Experience taught by nursing staff	7.70
	Teacher training by medical colleges or health vocational colleges	23.10
	Training of hospital or community medical service centers to pension institutions	6.70
	Professional training institutions	15.40
	Arrange for further study	15.40
	Others	1.90
Training contents	Psychological nursing knowledge	11.70
	Rehabilitation nursing knowledge	13.20
	Elderly people’ s daily life nursing knowledge	15.20
	First aid knowledge	10.30
	Basic nursing knowledge of common diseases of the elderly	9.40
	Safe handling knowledge	11.70
	Specialized disease nursing knowledge of the elderly	10.00
	Changing the concept of elderly care	7.30
	Humanistic care	10.30
	Others	0.90
Specialized disease nursing knowledge training	Hypertension	19.80
	Coronary heart disease	12.20
	Diabetes	20.90
	Stroke	15.70
	Senile dementia or mental illness	16.30
	Intensive care	11.00
	Others	4.10

### Analysis of comprehensive quality and sociodemographic characteristics relationship

The ownership of working unit was a significant predictor of professional knowledge, as community hospital achieved lower scores than nursing home (β = -0.207, *P* = 0.014). Education (β = 0.263, *P* = 0.002) and years of elderly rehabilitation nursing (β = 0.201, *P* = 0.005) also influenced their professional knowledge. The nursing staff’s gender also influenced their professional skills, with male nursing staff being better at it (β = 0.159, *P* = 0.021). The ownership of working unit was a significant predictor of professional skills, as geriatric hospital achieved higher scores than nursing home (β = 0.145, *P* = 0.038). The nursing staff with higher levels of education were more skilled at professional skills (β = 0.331, *P* = 0.000). Elderly rehabilitation nurses without practicing certificate were not good at professional skills (β = -0.296, *P* = 0.003). Also for showing comprehensive quality, the nursing staff’s gender (β = 0.149, *P* = 0.046), educational level (β = 0.293, *P* = 0.000) and years of elderly rehabilitation nursing were significant predictors (β = 0.172, *P* = 0.011) (Table 4).

**Table 4:** Linear Regression analysis for Comprehensive quality Subscale

Subscales	Characteristics	b	SEb	β	P
Professional Knowledge (R^2^ = 0.239)	Gender (M/F)	9.501	6.166	0.120	0.125
	Age (years)	0.424	2.231	0.017	0.850
	Community hospital (vs Nursing home)	-8.823	3.563	-0.207	0.014
	Education (Junior high school and below, …)	4.601	1.459	0.263	0.002
	Technical title (No, Nurse, Senior Nurse, …)	0.133	1.509	0.011	0.930
	Years of elderly rehabilitation nursing (years)	3.196	1.124	0.201	0.005
	Current monthly income (CNY)	0.210	1.245	0.013	0.866
	Nurse practicing certificate (no/yes)	-6.296	4.952	-0.145	0.205
Professional Skills (R^2^ = 0.421)	Gender (M/F)	7.109	3.064	0.159	0.021
	Age (years)	-0.305	1.121	-0.022	0.786
	Geriatric hospital (vs Nursing home)	4.398	2.099	0.145	0.038
	Health status (Health, …)	-3.911	2.205	-0.108	0.078
	Education (Junior high school and below, …)	3.277	0.720	0.331	< 0.001
	Technical title (No, Nurse, Senior Nurse, …)	-0.089	0.748	-0.013	0.905
	Current monthly income (CNY)	0.432	0.608	0.046	0.478
	Nurse practicing certificate (no/yes)	-7.267	2.440	-0.296	0.003
Professional Attitude (R^2^ = 0.051)	Gender (M/F)	0.523	0.924	0.050	0.572
	Age (years)	-0.295	0.337	-0.089	0.382
	Health status (Health, …)	-1.008	0.662	-0.119	0.130
	Education (Junior high school and below, …)	-0.017	0.216	-0.007	0.939
	Technical title (No, Nurse, Senior Nurse, …)	0.173	0.226	0.108	0.443
	Years of elderly rehabilitation nursing (years)	0.138	0.167	0.065	0.410
	Current monthly income (CNY)	0.190	0.188	0.087	0.313
	Nurse practicing certificate (no/yes)	0.051	0.735	0.009	0.944
Comprehensive Quality (R^2^ = 0.325)	Gender (M/F)	18.066	8.990	0.149	0.046
	Age (years)	0.434	3.279	0.011	0.895
	Community hospital (vs Nursing home)	-11.625	5.156	-0.178	0.025
	Health status (Health, …)	-12.059	6.443	-0.124	0.063
	Education (Junior high school and below, …)	7.822	2.107	0.293	< 0.001
	Technical title (No, Nurse, Senior Nurse, …)	-0.197	2.196	-0.011	0.929
	Years of elderly rehabilitation nursing (years)	4.195	1.623	0.172	0.011
	Current monthly income (CNY)	0.969	1.828	0.039	0.597
	Nurse practicing certificate (no/yes)	-13.542	7.156	-0.204	0.060

## Discussion

### The current situation of comprehensive quality of elderly rehabilitation nursing staff in medical and health care institutions

The results of this study showed that the overall score of the comprehensive quality of elderly rehabilitation nursing staff in medical and health care institutions is at a medium level. In each dimension, the highest score was professional attitude, followed by professional skills, and the lowest score was professional knowledge. These results are attributed to many reasons. Firstly, although in recent years, China has gradually attached importance to the training of elderly nursing professionals, but due to the late start, there is no specific theoretical knowledge system [18]. Secondly, the elderly nursing courses in China are mostly aimed at the teaching and training of professional knowledge of students at higher vocational and undergraduate levels, while there is a lack of standardized training and guidance for elderly nursing staff [19]. Through the questionnaire survey, it is known that most elderly rehabilitation nursing staff hope to learn systematic theoretical knowledge and skill operation through a variety of training methods. It was believed that the training courses for the elderly such as humanistic care and communication skills should be added, which could improve their comprehensive quality in all aspects in order to provide better humanistic care services for the elderly.

### The comprehensive quality in different types of elderly rehabilitation nursing staff in medical and health care institutions

Wang Xichen et al. [20] pointed out that nursing skills of elderly nursing staff in China are not equal with a big gap. Our results showed that female elderly rehabilitation nurses had better mastery of professional knowledge and professional skills compared to male (*P* < 0.01). It may be related to more women in this survey. Furthermore, most men are managers who are less familiar with professional knowledge and skills in comparison with clinical nurses. In addition, the mastery of professional knowledge of elderly rehabilitation nursing staff in the age group of 56 and above was the best, while the mastery of professional skills in the age group of 35 and below was higher than that in other age groups (*P* < 0.05). And in terms of technical title, the scores of professional knowledge of the co-chief superintendent nurses and above groups were the highest, while the scores of professional skills of the supervisor nurses group were higher than those of other technical title groups (*P* < 0.01). With the accumulation of working time, the elderly rehabilitation nursing staff in the senior age group were promoted to co-chief superintendent nurses and above professional titles. Through years of learning, they gradually mastered more professional knowledge of elderly nursing, which are generally deeply rooted. Elderly rehabilitation nursing staff aged 35 and below were the main force of clinical work because they were mainly in clinical work, and their technical titles were mostly supervisor nurses. Therefore, their mastery of clinical professional nursing skills would be more profound.

### Factors affecting comprehensive quality of elderly rehabilitation nursing staff in medical and health care institutions

#### Educational level

The results of this study showed that the comprehensive quality of elderly rehabilitation nursing staff in medical and health care institutions was positively correlated with educational level (*P* < 0.01). The comprehensive quality of elderly rehabilitation nursing staff with bachelor degree or above was significantly higher than that of other educational groups, indicating that the higher the educational level, the higher the comprehensive quality. In terms of dimensions, the scores of professional knowledge and professional skills in the bachelor degree or above education groups were significantly higher than those in other education groups (*P* < 0.01). It indicated that with the improvement of educational level, the cognitive level of professional knowledge was bound to improve, and more effective access to richer and more cutting-edge knowledge of elderly care. Moreover, with a stronger learning enthusiasm, they would be more familiar with the mastery of professional skills.

#### Years of elderly rehabilitation nursing

Our results suggested that the comprehensive quality of elderly rehabilitation nursing staff in medical and health care institutions was positively correlated with the number of years engaged in elderly rehabilitation nursing (*P* < 0.05). According to the group of years of elderly rehabilitation nursing, the scores of professional knowledge and professional skills of elderly rehabilitation nursing staff engaged in more than 10 years were significantly higher than those engaged in less than 1 year (*P* < 0.05). It indicated that with the increase of working years, the comprehensive quality would be improved accordingly. Meanwhile, the familiarity with professional knowledge and professional skills would be better with the increase of time.

### Countermeasures for improving comprehensive quality of elderly rehabilitation nursing staff in medical and health care institutions

#### Strengthen the training of elderly rehabilitation nursing professionals

At present, the training of elderly nursing professionals in China is still in its infancy. The elderly nursing professionals are far from enough to meet the needs of today' s society. The construction of nursing talent team is one of the bottlenecks for the long-term development of elderly nursing [21]. Therefore, it is urgent to cultivate high-quality elderly rehabilitation nursing professionals. However, the lack of health human resources has led to the lack of specialized medical, rehabilitation and health services in many medical and health care institutions [14]. Also, only《geriatric nursing》course is set up in clinical nursing specialty in colleges and universities in China, with fewer hours. So, students can only simply learn the basic knowledge of elderly care, and the training mode is relatively simple. Moreover, there is a lack of targeted practical skills of elderly care and opportunities for internships [22, 23].

A number of studies have pointed out that major colleges and universities can establish a cooperative relationship with medical and health care institutions, adopt a multi-teaching method, and formulate corresponding teaching objectives based on the different conditions of the elderly [23–25]. It is not only necessary for students to learn the theoretical knowledge of elderly care, but also create more practical opportunities for them to gradually adapt to the working environment of medical and health care institutions. Colleges and universities should also adopt interesting teaching methods to attract more students to fill in geriatric nursing-related majors and improve students ' interest in the actual teaching process [26].

#### Standardized training of elderly rehabilitation nursing professionals

The survey found that 52.90% of the elderly rehabilitation nursing staff spent most of their time working for disease professional care, followed by daily care, and only a small number of nursing staff spent most of their time working for psychological care and medication guidance for the elderly, which was similar to the findings of Liu Hui et al. [27]. When asked what aspects of their abilities should be improved, the research objects of this study indicated that their comprehensive quality should be comprehensively improved. They should not only learn advanced theories and skills, but also cultivate the communication ability with the elderly. They should also provide psychological guidance and give humanistic care to the elderly in life, help them improve their quality of life and reduce the existing physical and mental pain. Faria et al. [28] also emphasized that elderly rehabilitation nursing staff can better accompany the elderly by designing and implementing active aging programs for the elderly by focusing on lifestyle, balanced training and promoting social participation.

Yang Li et al. [29] found that 70.00% of managers in medical and health care institutions have received relevant training in their work units, 56.60% of managers have received provincial or municipal training, but few have received professional training at the international level. This is different from the results of this study, which may be related to the fact that the respondents in this study are mostly nursing staff rather than managers. Although the State Council has proposed to promote the development of the combination of medical and health care since 2013, the government has less opportunities to provide training for nursing staff. The vast majority of personnel said that their training knowledge is mainly the experience accumulated gradually in their work. A number of survey results show that many elderly care workers in medical and health care institutions have not participated in pre-job training courses [24, 30, 31], which will bring unknowable safety hazards to the elderly. This requires us to explore a set of training system suitable for elderly nursing staff in China and carry out various forms of training courses. Medical and health care institutions should also provide more training opportunities for elderly rehabilitation nursing staff to learn advanced concepts of elderly rehabilitation nursing. Gilissen el al [32] suggested on-the-job training for health care staff in nursing homes, standardized documentation, structured dialogue, and promotion of multidisciplinary awareness. Managers in the institutions should also actively participate in international and national training courses, absorb excellent management experience at home and abroad for the management of the institution.

#### Strengthen propaganda to eliminate inherent prejudice and improve the remuneration of elderly rehabilitation nursing staff

The results of this study showed that the actual monthly income of elderly rehabilitation nursing staff in medical and health care institutions is different from their expected monthly income, which is similar to the results of Lin and Zhang [17, 33]. For multiple reasons, such as low wages, low development space, and low social status of elderly care, it is difficult for professional medical staff or managers to work in the medical and health care institutions for a long time. It will seriously affect the service quality of medical and health care institutions and the development of the combination of medical and health care mode.

A number of studies have shown that, the elderly are more accustomed to family care, rather than institutional care, some elderly even feel sent to pension institutions is a ' shame ' [14, 34, 35]. The government should increase publicity through social, media and other channels to change the occupational prejudice of society and students regarding elderly care. Encourage more medical graduates to work in medical and health care institutions, and establish relevant incentive and subsidy policies to link salary treatment with vocational skill levels, so as to attract more outstanding elderly rehabilitation nursing professionals [23, 36]. Managers in the institutions should standardize the talent recruitment system, job title evaluation and continuing education work mechanism, and effectively protect the self-interest of elderly rehabilitation nursing staff. Corresponding welfare subsidies should also be given to mobilize their enthusiasm for employment. At the same time, the nursing staff in the institutions should be regularly provided with professional prospects and ability training to clarify their job orientation and expectations for the future. This will gradually improve their work enthusiasm and professional identity [20], and create a good and positive working environment for elderly rehabilitation nursing staff.

### Limitations

This study has several limitations. First, this study only investigated the comprehensive quality status of elderly rehabilitation nursing staff in Liaoning Province's medical and health care institutions at a certain point in time, and did not conduct a comparative study on them after participating in the training. Second, due to lack of human, financial and material resources, only 189 elderly rehabilitation nursing staff in Liaoning Province’s medical and health care institutions were surveyed, with a small sample size. Follow-up research can increase the sample size and expand the scope of the research, not limited to Liaoning Province, but extended to the medical and health care institutions across the country.

## Conclusions

The comprehensive quality of elderly rehabilitation nursing staff in medical and health care institutions in Liaoning Province is at a medium level. There is a lack of elderly rehabilitation nursing talents, and there is little opportunity to receive training in professional knowledge and skills related to elderly care. And the educational level of elderly rehabilitation nursing staff in medical and health care institutions and the years of elderly rehabilitation nursing are the main influencing factors of the comprehensive quality. It is suggested that relevant government departments should actively promote the construction of disciplines related to geriatric nursing and encourage graduates of relevant majors to work in medical and health care institutions. At the same time, the salary level and welfare benefits of the elderly rehabilitation nursing staff in medical and health care institutions should be appropriately increased. In addition, nursing managers and educators should formulate a scientific and reasonable training system in order to cultivate high-quality elderly rehabilitation nursing talents, which could provide better care services for the elderly in the institution and improve their quality of life.

## Data Availability

All data generated or analysed during this study are included in this published article.

## References

[1] Liu R (2019). Research and analysis of vocational training methods for elderly care. Think Tank Era.

[2] Yang J. Wang S. Liu Y. 70 Years of New China: An Analysis of the Development Trend of Population Aging. Chin J Popul Sci. 2019;04:30–42+126. (In Chinese).

[3] Wang Y (2020). Research on the innovative development path of the supply side of the integrated model of mental health and medical care in the new era: Taking Shenyang as an example.

[4] National Health and Family Planning Commission of the People's Republic of China. The General Office of the State Council forwards the notice of the Health and Family Planning Commission and other departments on the guidance of the promotion of the integration of medical and health services with elderly care services (Guo Fa Ban [2015] No. 84) [EB/OL]. (2015–11–20) [2016–11–18]. http://www.nhfpc.gov.cn/zhuz/xwfb/201511/6cb2dd9263d243fd8a031e635a21bce5.shtml. (In Chinese).

[5] Xing Y, Cheng S, Guan Y (2020). Research on the development of a new old-age care model of ‘combination of medical and elderly care’ from the perspective of nursing professionals. Chin Foreign Entrep.

[6] Li W, Wang L, Kang F (2019). Analysis of the current situation of the combination of medical and nursing development in china and its countermeasures. China Health Industry.

[7] Du N, Wu P, Yuan M (2021). Performance evaluation of combining with medical and old-age care in pension institutions of China: a two-stage data envelopment analysis. Risk Manag Healthc Policy.

[8] Zhou Y. Study on the Problems and Countermeasures of the Combination of Medical and Nursing Care in Chang Zhou. Xuzhou: China University of Mining and Technology. 2020. (In Chinese).

[9] Guo D. Li H. Li X. et al. The Feasibility of Serving the Elderly with the Combination of Medical Care. Int Med Health Guid News. 2005;21:45–46. (In Chinese).

[10] Xi Y, Zhang W, Li X (2019). Status quo analysis of the nursing professionals under the medical-nursing combined service. Chin Nurs Res.

[11] Wang P. Lei Y. Lv P. Analysis on the construction of a medical-care integration mechanism beyond multiple games: the dilemma and solution of my country's medical-care integration model. J Chin Acad Gov. 2018;02:40–51+135. (In Chinese).

[12] Du C, Zhang M, Yan Y (2017). Collaboration between colleges and universities to explore a new mode of training talents with integrated medical care and elderly care. Chin J Hosp Adm.

[13] Wei Y, Zhang L (2020). Analysis of the influencing factors on the preferences of the elderly for the combination of medical care and pension in long-term care facilities based on the Andersen model. Int J Environ Res Public Health.

[14] Zhang L, Zeng Y, Fang Y (2017). The effect of health status and living arrangements on long term care models among older Chinese: a cross-sectional study. PLoS One.

[15] Rezaei-Shahsavarloo Z, Atashzadeh-Shoorideh F, Gobbens RJJ (2020). The impact of interventions on management of frailty in hospitalized frail older adults: a systematic review and meta-analysis. BMC Geriatr.

[16] Wang C, Liu H, Wang Z (2019). Construction of old-age care knowledge system from perspective of combination of medical treatment and endowment. Chin Nurs Res.

[17] Lin S. Study on the quality of caregivers in institutions of elders care in Fujian Province. Hangzhou: Zhejiang University. 2019. (In Chinese).

[18] Yuan Y (2020). Investigation of nursing for the aged under the background of endowment pattern of “combination of medical treatment and endowment”. Heilongjiang Sci.

[19] Xing Q, Liu Y, Lin L (2020). Construction of training curriculum system on geriatric care among nurses on the job under the medical⁃nursing combined service backgrounds. Chin Nurs Res.

[20] Wang X, Lv X, Zhou L (2016). Research progress on training mode of endowment care workers from the perspective of the combination of medical and endowment model. Chin Nurs Manag.

[21] Zheng D, Xiao Y (2011). Thought on building the long-term care system for the elderly in china under the circumstances of new medical reform. Med Soc.

[22] Liu Y, Guo G (2011). The demand status of elderly nursing in my country and thinking on the training of elderly nursing talents. Chin Nurs Manag.

[23] Wang F (2020). Training of nursing talents for the aged in private colleges and universities under the mode of combination of medical and nursing. Heilongjiang Sci.

[24] Zhang Y, Li M, Wang L (2020). Research on the countermeasures for the construction of the long-term nursing service system for the elderly in Harbin. Decision-Making Consultancy.

[25] Shi X, Sun C, Qiu Y (2017). A study on the convergence of ‘3+3’ secondary and higher vocational courses for the elderly service and management majors——taking ‘elderly care and health care’ as an example. J Jiangsu Inst Commer.

[26] Hu J, An M (2020). Analysis of the status quo of elderly nursing personnel under the medical care model. Ind Innov.

[27] Liu H, Shen J (2010). Investigation and analysis of 298 clinical nurses' elder care ability. Guangdong Med J.

[28] Faria A, Martins M, Ribeiro O (2020). Elderly residents in the community: gaining knowledge to support a rehabilitation nursing program. Rev Bras Enferm.

[29] Yang L, Peng H, Yang Y (2020). Situation and countermeasures of the management team of the elderly care institutions from the perspective of the combination of medical and health care: a cross-sectional study. J Healthc Eng.

[30] Shi Y, Yu C, Liu H (2013). Survey of work and training and practicing status of nursing member in Wenzhou city. Chin Nurs Res.

[31] Li Y. Active Prevention and Response to Accidental Injuries of the Elderly in Elderly Care Institutions—Taking the Social Welfare Institute of Shaoxing City, Zhejiang Province as an Example. Social Welfare. 2016;(09):45–48+61. (In Chinese).

[32] Gilissen J, Pivodic L, Gastmans C (2018). How to achieve the desired outcomes of advance care planning in nursing homes: a theory of change. BMC Geriatr.

[33] Zhang T, Wang E (2020). Study on the status quo of nursing human resources in medical care institutions. China Rural Health.

[34] Fu Y, Guo Y, Bai X (2017). Factors associated with older people's long-term care needs: a case study adopting the expanded version of the Anderson Model in China. BMC Geriatr.

[35] Zhang L, Zeng Y, Wang L (2020). Urban-rural differences in long-term care service status and needs among home-based elderly people in China. Int J Environ Res Public Health.

[36] Chen Y, Chen L, Chen L (2015). Investigation on nursing homes’ requirements of older adults caregivers regarding their knowledge and core ability in Zhejiang province. J Nurs Sci.

